# Adaptive 3D Self‐Assembly of Colorectal Cancer Cells With Unchanged Tumor Phenotype and Drug Sensitivity

**DOI:** 10.1002/cam4.71017

**Published:** 2025-07-04

**Authors:** Yong Zhang, Yang Liu, Yang Yang, Yiwei Li, Lei Liu, Yang Huang, Weiting Zhao, Yong Liu, Minghao Zhang, Yunshan Zhao, Chenggang Li

**Affiliations:** ^1^ Senior Department of General Surgery, the First Medical Center of Chinese PLA General Hospital Beijing China; ^2^ Senior Department of Hepato‐Pancreato‐Biliary Surgery, the First Medical Center of PLA General Hospital Beijing China; ^3^ Darkjade Sciences, Inc. Beijing China; ^4^ Beijing Fengtai Hospital of Integrated Traditional Chinese and Western Medicine Beijing China

**Keywords:** adaptive remodeling, drug sensitivity, self‐assembly

## Abstract

**Background:**

Three‐dimensional (3D) self‐assembly of organoids or tumoroids based on 3D rebuilding environment (3DRE) aims to preserve the biological characteristics of original tumors, but whether the self‐assembly process is influenced by 3DREs remains unknown. We here compared the colorectal cancer (CRC) tumoroids cultured using different 3DREs, including dome culture (DG), ultra‐low adherence culture without Matrigel(UA) or with Matrigel (UAG), and hanging‐drop without Matrigel (HD) and with Matrigel (HDG).

**Methods:**

CRC cells were cultured to form tumoroids using DG, UA, UAG, HD, and HDG, respectively. The differences between these tumoroids were examined using light observation, histological staining, RNA sequencing, and drug sensitivity testing.

**Results:**

The CRC cells aggregated with each other and formed larger, converged tumoroids in the UA and the HD compared to the Matrigel. Histochemical examination revealed that the tumoroids maintained the CRC‐specific characteristics of forming lumens and biomarkers, but the number of lumens decreased, and the cell arrangement was in disorder with increasing impetus of 3DREs that promote cell aggregation ranging from DM, UA to HD. RNA sequencing revealed that the tumoroids retained a similar gene expression pattern, but the oncogenes related to metastasis and poor prognosis were upregulated, and development and morphogenesis‐related genes were downregulated when cultured in the UA and HD compared to the Matrigel. However, the drug sensitivity test showed that the tumoroids, regardless of the methods they were derived, maintained similar sensitivity to drugs.

**Conclusions:**

We demonstrated that CRC tumoroids developed towards disordered self‐assembly in adaptation to the environment without a matrix, but this adaptation did not alter tumor‐specific phenotypes and drug sensitivity.

**Abbreviations**
3DREs: three‐dimensional rebuilding environments
CRC: colorectal cancer
DG: dome culture
HD: hanging drop
HDG: hanging drop with Matrigel
UA: ultra‐low adherence
UAG: ultra‐low adherence with Matrigel

## 1 Background

Tumoroids that self‐assemble based on three‐dimensional rebuilding environments (3DREs) mimic the biological characteristics of original tumors, and are widely used as models for tumor biology research, drug screening, and drug sensitivity testing [1, 2, 3]. The 3DREs, which may disturb self‐assembly, resulting in altered tumoroids, are important factors that potentially influence the efficacy of models. In the past decades, several 3D culture strategies, including hanging drop (HD), ultra‐low adherence (UA), and embedment in Matrigel, which connote different 3DREs, have been established [2, 3, 4, 5, 6]. Tumoroids self‐assembled in Matrigel recapitulate genomic, DNA methylation, and transcriptome features of the original tissue, which is undoubtedly the gold standard for tumoroid culture [2, 3]. Panek et al. [5] confirmed that chicken embryo intestine organoids could be formed in HD. Human intestinal cells self‐assemble into organoids when cultured using UA, and the suspension culture can even promote serosal mesothelial development [6]. These results indicate that organoids or tumoroids can be self‐assembled using UA and HD, other than embedment in Matrigel. However, the impact of different 3DREs on the self‐assembly of tumoroids remains to be defined.

The distinguishing difference among these 3DREs is the impetus that promotes cell aggregation. Matrigel‐embedded culture, such as dome culture (DM), which mimics the natural matrix environment, provides a relaxing environment, and cells can self‐assemble as they do in vivo [3]. In contrast, the self‐assembly in HD and UA depends on the mutual cell aggregation, and floating cells are forced to aggregate with each other and form an integrated microtissue due to the deprivation of the surface that tumor cells can adhere to in a two‐dimensional culture system [5, 6]. The cell aggregation is further enhanced by the U‐shape and traction of gravity in the HD culture system [5]. Therefore, ranging from DM, UA to HD, the impetus of the 3DREs that promote cell aggregation increases sequentially. Additionally, embedding cells in Matrigel either in HD (HDG) or UA (UAG)is a complex 3DRE that affords cells a relaxing environment under the impetus niche. How the difference implicated in 3DREs influences the self‐assembly of tumoroids remains unknown.

Colorectal adenocarcinoma displays glandular tubular arrangement, and the tumoroids derived from adenocarcinoma recapitulate the structural characteristics with the formation of ductal structure or lumens [2]. Many oncogenic genes implicated in the development, invasiveness, and metastasis of colorectal adenocarcinoma are up‐regulated. ETS1 has been reported to be directly involved in the invasion and metastasis of colorectal cancers [7]. C14orf169 is selectively expressed in CRC tissues and is involved in metastatic phenotypes [8]. FAM203B (HGH1) mediates colorectal cancer cell proliferation, migration, EMT, and stemness [9]. In the present study, we compared the tumoroids that self‐assembled in different 3DREs, including DG, UA, UAG, HD, and HDG, and the impacts of 3DREs on the tumoroids were analyzed.

## 2 Results

### 2.1 Adaptive 3D Self‐Assembly of Tumoroids

**FIGURE 1:**
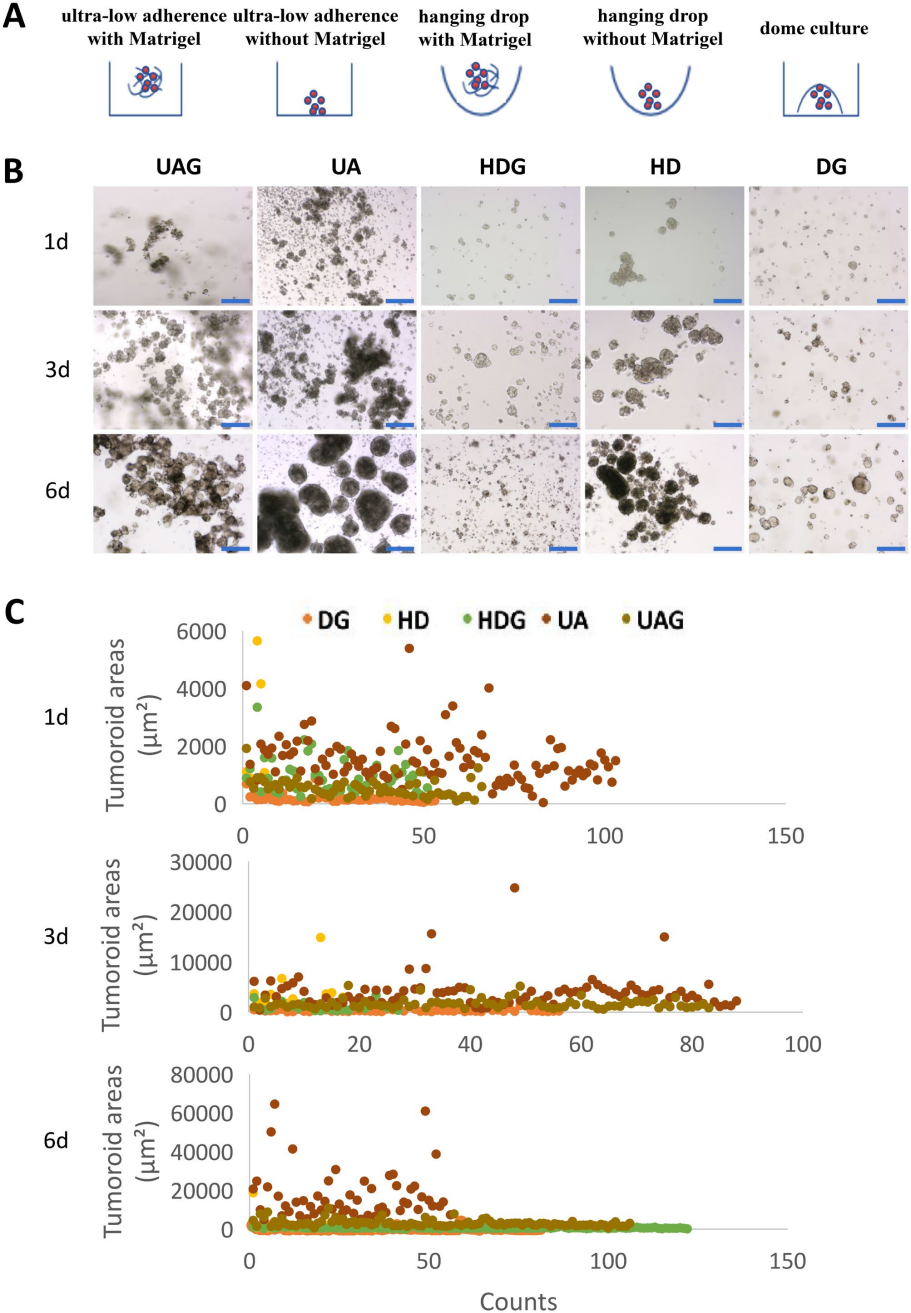
Self‐assembly of CRC cells in different 3DREs. A. Schematic representation of the 3D culture systems: Ultra‐low adherence culture with Matrigel (UAG); ultra‐low adherence culture without Matrigel (UA); hanging drop culture with Matrigel (HDG); hanging drop culture without Matrigel (HD); dome culture (DG). B. Representative images of CRC tumoroids cultured using UAG, UA, HDG, HD, and DG, respectively, showing that the sizes of tumoroids, which were various from 3DREs, increased over time, bar = 200 μm. C. Scatter plots of tumoroid sizes on day 1, day 3, and day 6, showing the distribution of tumoroid sizes during culture, and the ones in DG and UDG increased gradually, while the ones in HD and UA increased rapidly due to tumoroid aggregation.

A total of three colorectal adenocarcinoma samples were used to establish tumoroids individually. The CRC cells derived from the P3 tumoroids were cultured using DG, UA, UAG, HD, and HDG, respectively (Figure 1A). The cells in all groups self‐assembled into 3‐dimensional tumoroids whose sizes were various from 3DREs on the second day (Figure 1B). Uniform sizes of tumoroids were formed when they were embedded in Matrigel, including DG, UAG, and HDG, and large and converged tumoroids were generated due to aggregation when cultured in a floating condition without Matrigel, including HD and UA (Figure 1B,C). The sizes of tumoroids in all groups increased over time. The tumoroids in DG and UDG increased gradually, whereas those in HD and UA increased rapidly due to tumoroid aggregation (Figure 1B,C). By day 6, various sizes and shapes of the tumoroids were formed in HD and UA, and the largest area was over 60000μm^2^ (Figure 1C). In contrast, smaller and relatively uniform sizes of tumoroids were formed in DG and UAG (Figure 1C). Exceptionally, the sizes of tumoroids in the HDG increased in the first 3 days, but not thereafter (Figure 1B,C).

### 2.2 Retainment of Morphological Characteristics With Decreased Ability of Forming Lumens

**FIGURE 2:**
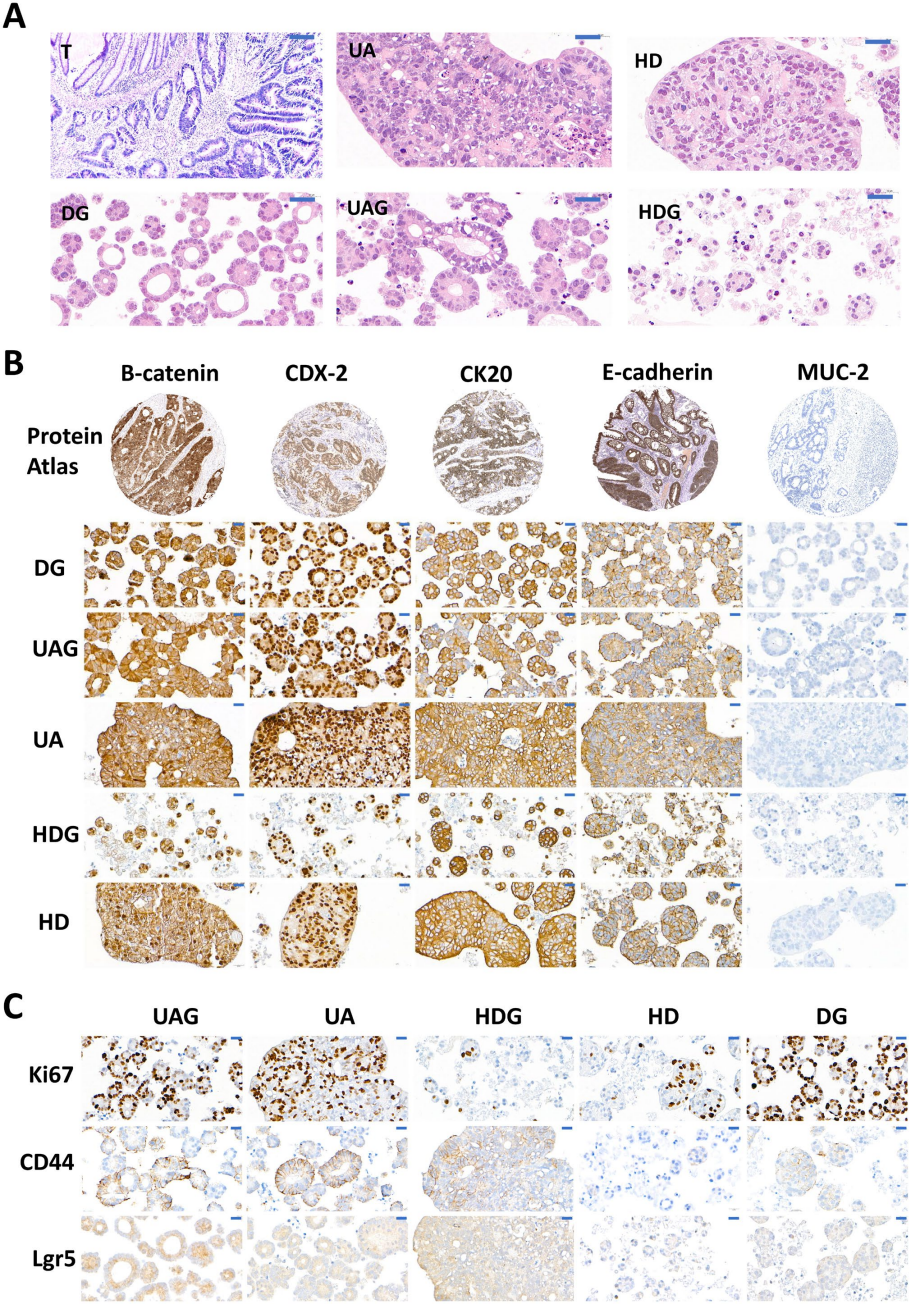
Morphology of parental tumor tissue (T) and tumoroids cultured using UAG, UA, HDG, HD, and DG. A. Representative H&E staining of parental tumor tissue and tumoroids, showing that the tumoroids displayed morphological characteristics of adenomatoid tumors with the formation of lumens whose morphology and numbers were variable from culture methods, bar = 20 μm. B. Immunohistochemical staining of tumoroids for E‐cadherin, β‐Catenin, CDX‐2, CK20, and MUC‐2 that were used as markers of colorectal cancers in the Protein Atlas database, showing that the expression pattern of these proteins was similar in all groups of UAG, UA, HDG, HD, and DG. Bar = 20 μm. C. Immunohistochemical staining of tumoroids for Ki67 and stem cell biomarkers of CD44 and lgr5, showing the differential proliferation of tumoroids from UAG, UA, HDG, HD, and DG and the existence of tumor stem cells in tumoroids, bar = 20 μm.

The tumoroids displayed morphological characteristics of adenomatoid tumors with the formation of lumens mimicking the original tumor (Figure 2A). The tumoroids embedded in Matrigel, including HDG or DG and UAG, were arranged in order and formed typical lumen structures (Figure 2A). However, with increasing impetus that promoted cells to aggregate, ranging from DG, HA to HD, the tissue density of tumoroids increased, while the number of lumens decreased, and the lumens were occasionally visible in the dense tissue in HD (Figure 2A). Immunohistochemical staining showed that the tumoroids from all the groups were positive for β‐Catenin, CDX‐2, CK20, and E‐Cadherin, and negative for MUC‐2, which were the featured markers of colorectal cancers, as shown in the Protein Atlas database (Figure 2B). Detection by staining against Ki67 demonstrated that the tumoroids displayed strong proliferation ability in DG, UA, and UAG (Figure 2C). However, the proportion of proliferated cells in hanging drops with or without Matrigel was distinctly low (Figure 2C). We also detected tumor stem cells by staining CD44 and Lgr5 markers. Our results demonstrated the existence of tumor stem cells in all groups, but the ratio was much lower in HDG than in other groups (Figure 2C).

### 2.3 Maintenance of Gene Expression Pattern With Increased Oncogene Expression and Decreased Morphogenesis Gene Expression

**FIGURE 3:**
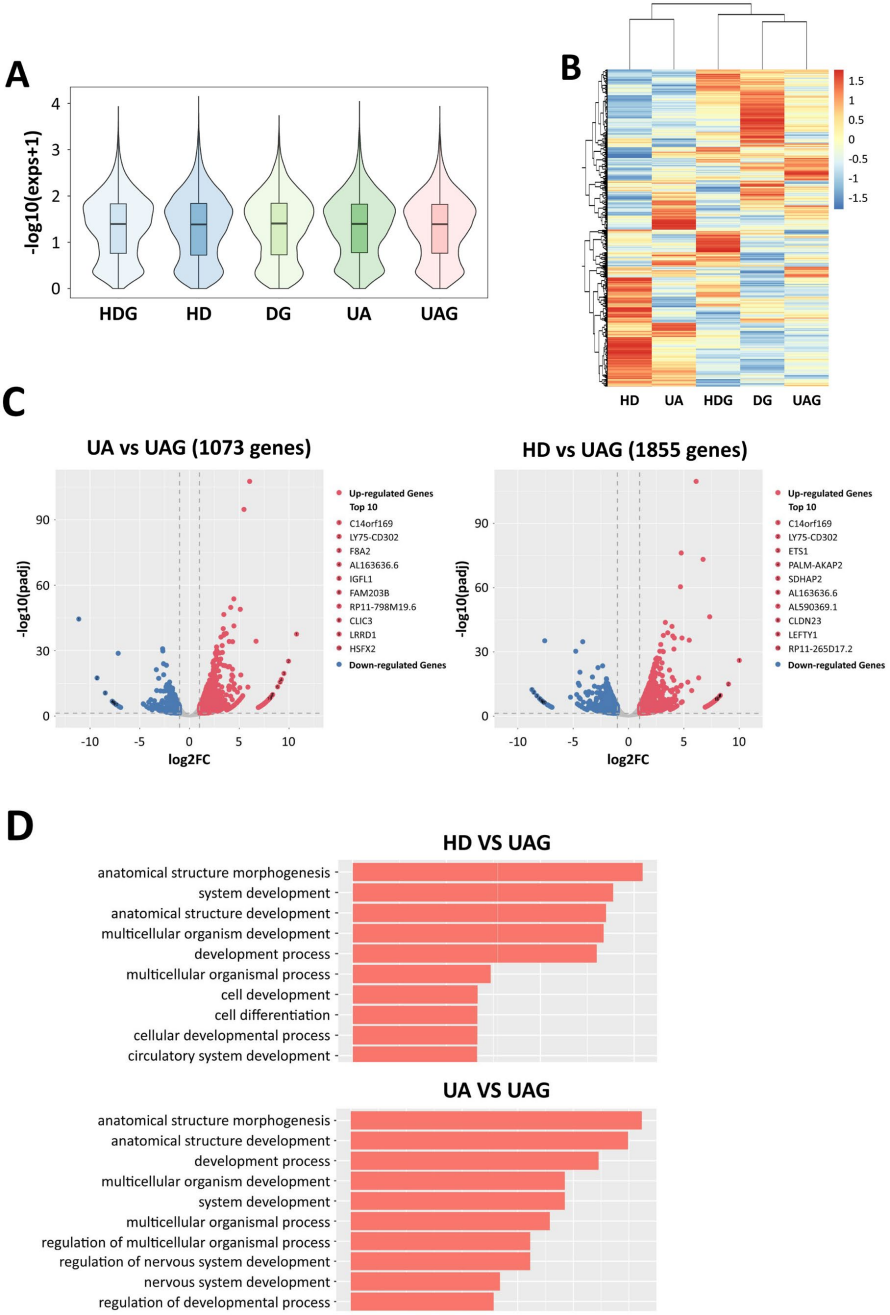
Transcriptomic profiling of tumoroids cultured using UAG, UA, HDG, HD, and DG. A. Violin plots, showing that the tumoroids cultured using UAG, UA, HDG, HD, and DG displayed similar gene expression patterns. B. Gene cluster analysis revealed differential gene expression of tumoroids related to the 3DREs. C. Differential gene expression analysis, showing that the up‐regulated genes were related to the malignant potential of tumors. D. GO enrichment analysis, showing that the differentially down‐expressed genes were significantly enriched in the development and morphogenesis.

RNA sequencing was performed on the tumoroids cultured using UAG, UA, HDG, HD, and DG, respectively. Violin plot showed that the tumoroids from UAG, UA, HDG, HD, and DG displayed similar gene expression patterns (Figure 3A). However, gene cluster analysis revealed that the tumoroids displayed hierarchical gene expression related to the 3DREs, and the tumoroids embedded in Matrigel, including DG, UAG, and HDG, had similar gene expression values, while the ones floating in medium without Matrigel, including HD and UA, shared similar gene expression (Figure 3B). To further reveal the differences among them, differential gene expression analysis was performed. Compared to UAG, most of the up‐regulated genes ranking top 10 were related to malignant potential of proliferation, progression, and tumor metastasis in HD and UA (Figure 3C). GO enrichment analysis showed that the differentially down‐regulated genes (HD versus UAG and UA versus UAG) were significantly enriched in the development and morphogenesis (Figure 3D).

### 2.4 Implication of Wnt Signaling in the Adaptive Morphological Alteration

**FIGURE 4:**
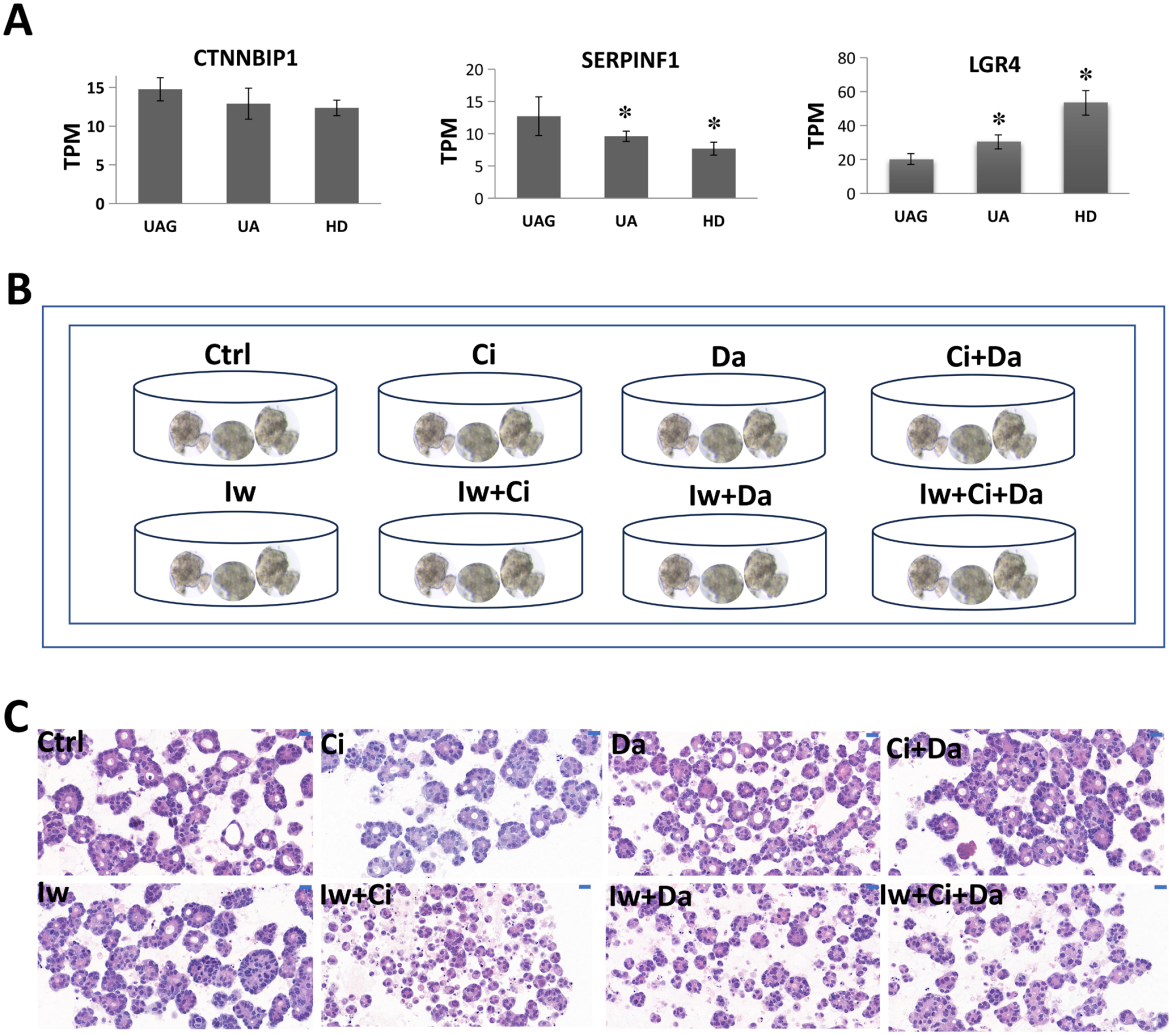
Cell signaling in regulating the morphogenesis of tumoroids. A Differential expression histogram (TPM: Transcripts Per Million) of CTNNBIP1, SERPINF1, and LGR4 implicated in Wnt signaling between UA or HD and UAG, showing the down‐regulation of CTNNBIP1 and SERPINF1, and up‐regulation of LGR4 between UA or HD and UAG (*n* = 3). ✽, *p* < 0.05. B Experimental design. The CRC tumoroids were treated with different signal transduction inhibitors of IWR‐1(Iw) for Wnt signaling, DAPT (Da) for Notch signaling, Ciliobrevin A (Ci) for Hedgehog signaling, and their combination. C, Representative H&E staining of tumoroids treated by signal transduction inhibitors of Iw, Da, Ci, Iw + Da, Iw + CI, Da + Ci, and Iw + Da + Ci, showing that Iw inhibited the formation of lumens. Bar = 50um.

Studies have shown that Wnt signaling regulates CRC development and morphogenesis. We therefore investigated whether the Wnt signaling played important roles in regulating the morphogenesis related to the 3DREs. Gene expression analysis demonstrated that the genes, catenin beta interacting protein 1 (CTNNBIP1) and serpin family F member 1 (SERPINF1), which negatively regulate the Wnt signaling activity, were down‐regulated, whereas the gene, Leucine‐rich repeat‐containing G protein‐coupled receptor 4 (LGR4), which positively regulates the Wnt signaling activity, was up‐regulated in HD and UA compared with UAG (Figure 4A). Further experiments confirmed that Wnt signaling inhibitor IWR‐1 inhibited the formation of lumens. In contrast, the Notch signaling inhibitor DAPT and the Hedgehog signaling inhibitor Ciliobrevin A did not influence the formation of lumens (Figure 4B,C).

### 2.5 Inalterable Response to Drugs

**FIGURE 5:**
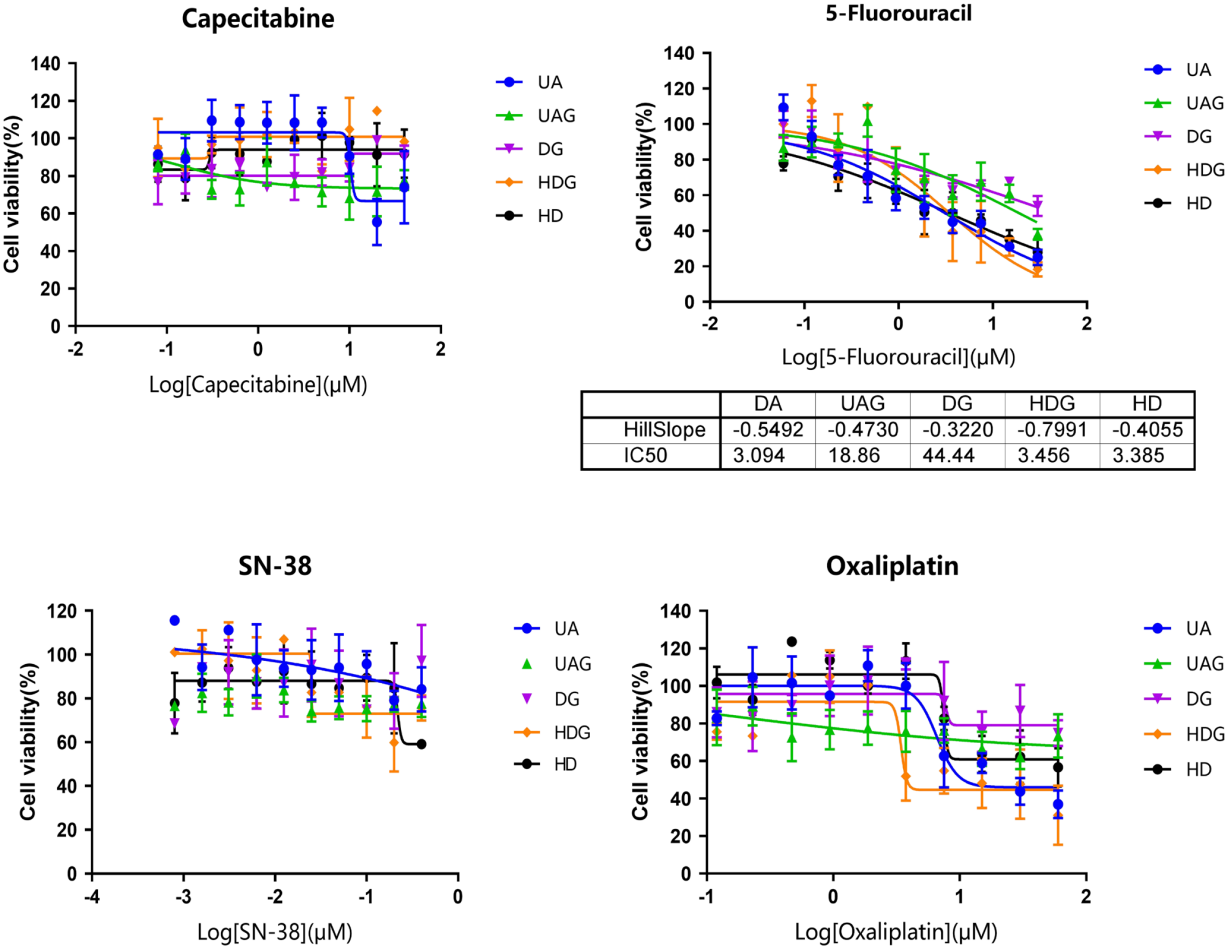
Dose–response curves of the tumoroids cultured using UAG, UA, HDG, HD, and DG after treatment with 5‐Fu, Capecitabine, S‐83, and Oxaliplatin, respectively (*n* = 3), showing that the tumoroids from whatever methods were derived displayed similar response to drugs regardless of different sizes.

To test the drug sensitivity of tumoroids cultured using UAG, UA, HDG, HD, and DG, we examined the responses of tumoroids to chemotherapeutic drugs including 5‐Fu, Capecitabine, S‐83, and Oxaliplatin. The tumoroids from HDG, HD, DG, UA, and UAG displayed similar dose–response curves despite different sizes and morphology (Figure 5).

## 3 Discussion

In the present study, we demonstrated that CRC tumoroids displayed adaptive self‐assembly to 3DRMs while maintaining characteristics of colorectal adenomatoid tumors. With the impetus that promoted cell aggregation, the CRC tumoroids developed towards disorderly characteristics as demonstrated by disorganized arrangement, upregulated cancer‐related genes, and downregulated development‐related genes. However, the adaptation, at least in a short time, did not alter cell phenotype and drug sensitivity.

Accumulating evidence demonstrated that CRC tumoroids recapitulated parental tumor features, including adenomatoid tumors, when they were embedded in Matrigel [2, 10, 11]. Consistently, our study showed that CRC tumoroids maintained the basic characteristics of colorectal adenomatoid tumors when cultured either in Matrigel or in HD and UA. This was supported by the finding that lumens could be formed in the tumoroids cultured using HD and UA. We further demonstrated that the tumoroids maintained cell phenotypes as revealed by immunohistochemical staining. CD44 and Lgr5 were surface markers of colorectal cancer stem cells, which played important roles in colorectal cancer metastasis and progression [12]. A previous study showed that spheroids were mainly composed of stem cells when cultured in a floating condition [13]. Detection by staining against CD44 and Lgr5 demonstrated that all the tumoroids contained tumor stem cells, indicating that tumor stem cells in tumoroids were not influenced by 3DREs in our study. Additionally, we also showed that the tumoroids had proliferative potential as demonstrated by immunohistochemical staining against Ki67 in UA, UAG, and DG. It was noteworthy that the ratio of proliferating cells was relatively low in HD and HDG compared to other groups, and this may be due to the lack of nutrition by the end of culture.

Furthermore, we demonstrated that the tumoroids displayed differential developmental patterning in adaptation to different 3DREs, as shown in Figure 2. The tumoroids embedded in Matrigel well displayed the characteristics of adenomatoid tumor and self‐assembled in order, and expressed more development‐related genes. In contrast, the tumoroids cultured in HD and UA were arranged in a disordered manner, and rarely formed luminal structures that adenomatoid tumors have due to the lack of anchoring sites in adaptation to the driving aggregation. Accordingly, most of the top 10 up‐regulated genes, such as C14orf169 and ETS1, were related to proliferation and metastasis [14, 15], indicating the increase of their malignant degree. Another gene of LY75‐CD302 in the top 10 up‐regulated genes was implicated in EMT [16]. Consistent with this, the genes of development and organ genesis that drove cell development and arrangement were down‐regulated in HD and UA. These results indicated that the CRC tumoroids developed towards disorderly self‐assembly in adaptation to the environment without a matrix.

Studies showed that the Wnt/β‐catenin signaling pathway was a complex network of protein interactions that functions most commonly in embryonic development and cancer [17, 18]. The genes CTNNBIP1 and SERPINF1 negatively regulated the Wnt signaling activity, while the gene LGR4 positively regulated it [19, 20, 21]. In colorectal cancer, the Wnt/β‐catenin signaling pathway was implicated as the central mechanism that drives colorectal carcinogenesis [17]. In our study, we showed that the expression of CTNNBIP1 and SERPINF1 was down‐regulated and the expression of LGR4 was up‐regulated in the HD and UA compared to the UAG, showing the adaptive response of the colorectal cancer cells to the 3DRMs that lacked matrix support and the efforts of improving the Wnt/β‐catenin signaling activity. Inhibition of the Wnt/β‐catenin signaling pathway, with decreased formation of lumens, further indicated that the signaling was implicated in the formation of lumens.

3D microtissue‐based drug test provided an effective method for predicting the sensitivity of tumor cells to anti‐cancer drugs [2]. Organoids or tumoroids that were cultured in the HD or UA had been used as microtissue for the test of drug sensitivity, but whether these methods had sensitive differences in response to drugs remained unknown. Our results showed that the tumoroids from whatever methods were derived displayed a similar response to drugs, indicating that the alterations that adapted to 3DREs did not influence the response to drugs.

## 4 Conclusions

We showed that colorectal cancer tumoroids exhibited adaptive remodeling to 3DREs during self‐assembly, with disordered arrangement and gene expression appearing in adaptation to an unstable environment. However, this adaptive alteration to 3DREs did not influence cell phenotypes and cell sensitivity to drugs, at least in the short term. Our study indicated that organoids or tumoroids cultured in different 3D rebuilding environments should be examined for their similarity before they are used for any experiment.

## 5 Methods

### 5.1 Statement of Compliance With Internal Review Boards

All methods were carried out according to relevant guidelines and regulations. Studies involving human tissues were approved by the Institutional Review Board (IRB, S2022‐148‐01). Informed consents were obtained from the patients whose tissue was used in our study.

### 5.2 Establishment of Tumoroid Cultures

Three colorectal adenocarcinoma samples were used for the assembly of tumoroids, respectively. Fresh colorectal tumor tissue was rinsed with advanced DMEM/F12 (AdDMEM, BasalMedia, Beijing, China) containing 1xpenicillin/streptomycin (P/S) ten times, and then was minced using scissors in a 6 cm dish with digestion buffer (AdDMEM containing 1XP/S, 2.5% FBS, 0.6 mg/mL collagenases, 20 mg/mL hyaluronidase). Tumor cells were released from bulk tissue by pipetting up and down several times, and the mixture was passed through a 100 μm cell strainer to remove tissue debris. After being washed twice using AdDMEM medium by centrifugation at 300 g for 5 min, cell clusters were resuspended in basal culture medium, Matrigel was added, and plated in a 24‐well ultra‐low adherence plate. After solidification, culture medium was added. The basal culture medium was modified from the literature [2] as follows: AdDMEM/F12 was supplemented with antibiotic‐antimycotic (BasalMedia, Beijing, China), 1 × B27(invitrogen/Gibco), 2 mM GlutaMAX, 10 nM gastrin I, 10 mM HEPES, 1 mM N‐acetylcysteine, and 10 mM nicotinamide (SigmaAldrich), 50 ng/mL human recombinant EGF (Peprotech), 500 nM A83–01 (Tocris, Avonmouth, Bristol, UK), and 10 μM SB 202190 (SigmaAldrich), 0.1 mg/mL R‐spondin, 0.1 mg/mL Noggin, and 0.1 mg/mL Wnt‐3A. The culture medium was changed every 2–3 days.

### 5.3 3D Culture Using Different 3DREs

CRC cells from P3 tumoroids were resuspended in basal culture medium or Matrigel. A drop of 30ul CRC tumor cells/Matrigel complex was plated onto the bottom of a tissue culture plate to generate dome culture (DG). For hanging drop culture, a small aliquot of cell suspension with or without Matrigel was introduced onto the surface of a 10 cm dish lid, and CRC cells grew in the hanging drop liquid with Matrigel (HDG) or without Matrigel (HD). For ultra‐low adherence culture, CRC cells with or without Matrigel were plated onto 24‐well plates, and the cells grew with Matrigel (UAG) or without Matrigel (UA). The cells with Matrigel were incubated at 37°C, 5% CO_2_ for 30 min after plating, and then culture medium was added. For the experiment of cell signaling inhibitors, the tumoroids cultured in Matrigel (UAG) were treated with 10 μM IWR‐1, 10 μM DAPT, and 10 μM Ciliobrevin A, respectively. All the cells were observed under an inverted microscope, and the images of tumoroids were captured using the Mshot image analysis system every day. The sizes of tumoroids were calculated as area.

### 5.4 Histological Examination

Tumor tissue or tumoroids were fixed with 10% neutral formalin overnight, paraffin‐embedded, and sectioned (4–5 μm). Sections were deparaffinized and hydrated, then stained with H&E for histological analysis. For immunohistochemical staining, sections were carried out using the following primary antibodies: anti‐E‐cadherin (Origene), anti‐β‐Catenin (Origene), anti‐CDX‐2(Origene), anti‐CK20(Origene), MUC‐2 (Origene), anti‐Ki67 (Origene), anti‐CD44 (Origene), and anti‐Lgr5 (Origene). All secondary antibodies were used at 1:500. All images were captured using CaseViewer.

### 5.5 RNA‐Sequencing Data Analysis

Tumoroids from DG, UA, UAG, HD, and HDG were collected 6 days after culturing. Total RNA was isolated using the RNeasy MinElute Cleanup kit (QIAGEN) according to the manufacturer's protocol. Quality and quantity of isolated RNA were checked and measured with the Agilent Bioanalyzer and Qubit 4.0, respectively. Library preparation was conducted with 1 μg of total RNA using the VAHTS Universal V6 RNA‐seq Library Prep Kit, and checked with Qubit dsDNA HS Assay Kit (Thermofisher) and Agilent D1000 Reagents. Libraries were equimolarly pooled to 2 nM and sequenced on the Illumina NovaSeq 6000.

### 5.6 Drug Treatment

Tumoroids cultured using DG, UA, UAG, HD, and HDG for 3 days were transferred to the wells of a 96‐well plate and allowed to recover for 2 to 3 days. Drugs, including 5‐Fluorouracil (5‐Fu), S‐38, capecitabine, and Oxaliplatin, were added, respectively, and cultured for another 3 days. 0.1% DMSO was used as a negative control, and Staurosporine was used as a positive control. At the end of treatment in each experiment, the number of viable cells was determined by CellTiter‐Glo assay (Promega, Madison, WI, USA) following the kit protocol. The data was compared with a positive control (1uM Staurosporin) and a negative control (DMSO). Dose‐response curves were generated using GraphPad Prism 8 (La Jolla, CA, USA).

### 5.7 Data Analysis

All values are presented as mean ± SE. All statistical analyses were performed using GraphPad Prism software (GraphPad 8.0). Sample size (n) values used for statistical analyses were provided in the relevant figures. Significance was set at *p* < 0.05.

## Author Contributions

**Yong Zhang:** conceptualization, writing – original draft, data curation. **Yang Liu:** conceptualization, data curation. **Yang Yang:** data curation. **Yiwei Li:** data curation. **Lei Liu:** investigation, methodology. **Yang Huang:** investigation, methodology. **Weiting Zhao:** data curation. **Yong Liu:** data curation. **Minghao Zhang:** data curation. **Yunshan Zhao:** conceptualization, investigation, methodology, writing – review and editing, writing – original draft, validation, data curation, formal analysis, project administration, supervision, resources, visualization. **Chenggang Li:** conceptualization, writing – review and editing, validation, software.

## Ethics Statement

Studies involving human tissues were approved by the Institutional Review Board of the First Medical Center of PLA General Hospital (IRB, S2022‐148‐01).

## Consent

The authors have nothing to report.

## Conflicts of Interest

The authors declare no conflicts of interest.

## Data Availability

The data that support the findings of this study are available on request from the corresponding author. The data are not publicly available due to privacy or ethical restrictions.
